# Open Frontiers in Neural Cell Type Investigations; Lessons From *Caenorhabditis elegans* and Beyond, Toward a Multimodal Integration

**DOI:** 10.3389/fnins.2021.787753

**Published:** 2022-03-07

**Authors:** Georgia Rapti

**Affiliations:** Developmental Biology Unit, European Molecular Biology Laboratory, Heidelberg, Germany

**Keywords:** neurons, glia, development, evolution, transcriptomics, genetics, databases, integration

## Abstract

Nervous system cells, the building blocks of circuits, have been studied with ever-progressing resolution, yet neural circuits appear still resistant to schemes of reductionist classification. Due to their sheer numbers, complexity and diversity, their systematic study requires concrete classifications that can serve reduced dimensionality, reproducibility, and information integration. Conventional hierarchical schemes transformed through the history of neuroscience by prioritizing criteria of morphology, (electro)physiological activity, molecular content, and circuit function, influenced by prevailing methodologies of the time. Since the molecular biology revolution and the recent advents in transcriptomics, molecular profiling gains ground toward the classification of neurons and glial cell types. Yet, transcriptomics entails technical challenges and more importantly uncovers unforeseen spatiotemporal heterogeneity, in complex and simpler nervous systems. Cells change states dynamically in space and time, in response to stimuli or throughout their developmental trajectory. Mapping cell type and state heterogeneity uncovers uncharted terrains in neurons and especially in glial cell biology, that remains understudied in many aspects. Examining neurons and glial cells from the perspectives of molecular neuroscience, physiology, development and evolution highlights the advantage of multifaceted classification schemes. Among the amalgam of models contributing to neuroscience research, *Caenorhabditis elegans* combines nervous system anatomy, lineage, connectivity and molecular content, all mapped at single-cell resolution, and can provide valuable insights for the workflow and challenges of the multimodal integration of cell type features. This review reflects on concepts and practices of neuron and glial cells classification and how research, in *C. elegans* and beyond, guides nervous system experimentation through integrated multidimensional schemes. It highlights underlying principles, emerging themes, and open frontiers in the study of nervous system development, regulatory logic and evolution. It proposes unified platforms to allow integrated annotation of large-scale datasets, gene-function studies, published or unpublished findings and community feedback. Neuroscience is moving fast toward interdisciplinary, high-throughput approaches for combined mapping of the morphology, physiology, connectivity, molecular function, and the integration of information in multifaceted schemes. A closer look in mapped neural circuits and understudied terrains offers insights for the best implementation of these approaches.

## Introduction: Nervous System Complexity and the Demand for Cell Classification

Neural circuits have long appeared resistant to a coherent reductionist understanding, partly due to their structural and functional complexity. Neuron numbers are high across species, from billions in human brains to millions in mouse and zebrafish brains, hundred thousand in *Drosophila melanogaster* and hundreds in *Caenorhabditis elegans*. Numbers of macroglia, neurons’ ectoderm-derived sister cells, rise from thousands to millions across vertebrates and dozens to thousands in invertebrate models. Neural cells have diverse properties delineating complementary perspectives; morphology (pattern of membrane projections), molecular features (neurotransmitter receptors, transporters, effector proteins), circuit function (chemosensory/mechanosensory/interneurons, myelinating/non-myelinating glia, etc.) (Zeng and Sanes, 2017; Allen and Lyons, 2018; Singhvi and Shaham, 2019; Bittern et al., 2020). It is well accepted that neural cell types serve as building blocks of circuits and dissecting their diversity and connectivity is key to investigate nervous system function.

Due to their diversity and sheer numbers, analyzing neural cells systematically requires categorizing them molecularly and functionally. Such classification serves various purposes. First, it allows experimental reproducibility; understanding nervous system biology requires consistent accessibility of defined cells across time and space, to allow coupling of their developmental programs to their functional roles. The resulting reduced dimensionality serves the need to interpolate information, assess known and unknowns, highlight emerging concepts, regulatory programs, functional mechanisms and evolutionary relations. As discussed below, in *C. elegans*, reliable identification of nervous system cells at single-cell resolution allows mapping of their connectivity and mechanistic understanding of their development and interactions. Gene-function discovery in cells with similar functions and molecular content dissects the disease mechanisms altering specific cells or genes across cell types (Takano, 2015; Ponroy Bally and Murai, 2021). Classification by criteria shared across organisms allows to evaluate knowledge in different models and to proceed in testable hypotheses. By investigating cell behavior and function across species, organisms may be understood in light of the cell types they present or lack (Marioni and Arendt, 2017).

Cell classification previously hampered by laborious approaches lacking quantitative reproducibility is fast becoming an issue of the past, resolved by recent high-throughput methods. Nevertheless, classifications are arbitrary man-made concepts; we compose categories while natural selection may be working toward continuums of diversity. Each cell type exists in a single state at a time, transitions between states in time and space, and can be thought of as a subset of cell states in a multidimensional space (Trapnell, 2015). Classification in the nervous system meets conceptual challenges; how fine or firm are the distinctions of cell types is difficult to define. Everyone agrees on broad classes of motorneurons and interneurons, astrocytes and oligodendrocytes, yet such coarse distinctions bear little use for the above-mentioned purposes. If each neural cell type differs from another in molecular, morphological, and functional properties combined, the conceptual challenge persists beyond the growing large-scale approaches that enable in-depth characterization of individual cells. Can we devise classification schemes or information arrangements that fairly balance overarching distinctions of cell types and within-cell-type variability? Cell types are defined by the possible space of their states, arising from an array of experimental descriptions recounting a cell’s content, development, and function. The aim of a classification in a given system (developmental, molecular, evolutionary neuroscience) should be clear, while if it is meant to serve multiple purposes, a multi-faceted and dynamic classification is key. A closer look at *C. elegans*, the first metazoan with nervous system anatomy, connectivity and molecular content mapped with single-cell resolution, highlights aspects of multi-faceted classification, providing lessons for workflows and challenges of such integration.

This review reflects on neural cell type classification and how recent research, in *C. elegans* and beyond, can guide nervous system study through integrated classification schemes. It does not intend to comprehensively summarize the *C. elegans* nervous system regulatory mechanisms or functions, reviewed elsewhere comprehensively (Hobert, 2016a). I discuss how gene-function analysis and recent advances in molecular atlases highlight unforeseen cell heterogeneity and classification challenges. I suggest integrated classification schemes in unified platforms to allow equal annotation of large-scale datasets with published or unpublished findings and community feedback. Altogether, using examples in and outside *C. elegans* research, I discuss how reconciling morphological, molecular, functional knowledge and classification approaches enables comprehensive nervous system study.

## Investigating Nervous System Cells Across Different Eras and Classifications

### Navigating From Cell Morphology to Activity

**FIGURE 1:**
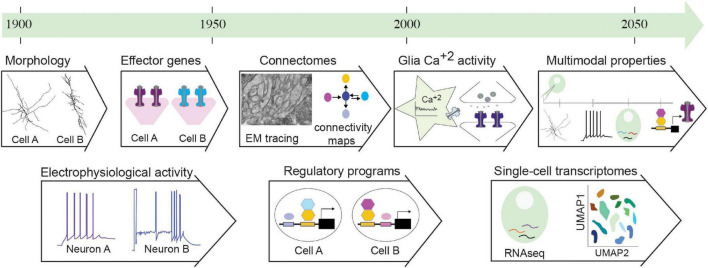
Nervous system properties serving cell classification criteria throughout time. This historical timeline presents properties of nervous system cells that served as classification criteria throughout the 20th and 21st centuries. Initial classifications followed morphological features. These were paired with electrophysiological recordings of neurons, but not glia. Soon, the revolution of molecular biology and genetics in model organisms uncovered effector genes of functional modules and regulatory programs of neural cells. Electron microscopy (EM) tracing enabled mapping synaptic connections, providing circuit connectomes. Recently, calcium activity was described in glial cells, implicated in regulating neuronal function. In the last two decades, transcriptomics and specifically single-cell transcriptomics enables classification of transcriptomic cell clusters through dimensionality reduction analysis (UMAP). Combination of these approaches pave the way to multimodal analysis of properties for integrated classifications. Details and relevant citations are found in the text. EM figure kindly provided by G. Rapti, Y. Lu, S. Shaham (unpublished data).

Cell type descriptions transform alongside our ever-progressing knowledge, within the nervous system and beyond. The first cell description was based on form; Hooke referred to “pores, or cells…”, due to the rigid wall of plant cells (Hooke, 1665). Two centuries later, Schultze casts aside this previous definition to define cells by their content and not their boundary; a “naked speck of protoplasm with a nucleus”(Kutschera, 2011). In 1896, Wilson described cells as “the basis of the life of all organisms”(Hyman and Simons, 2011). Similarly, nervous system cells were initially defined by morphology and architecture, and later functionally and molecularly (Figure 1). Ramoìn y Cajal provided one of the founding nervous system descriptions and the first extensive neuron classification based on morphology, the principal criteria available at the time (Ramón Y Cajal, 1911). Early drawings by Virchow and Deiter described the cells known today as (macro)glia, which were grouped morphologically by Lenhossek, Andriezen, and Koelliker in a classification largely adopted and developed by Cajal (García-Marín et al., 2007). Neurons and glia are now recognized cell components of all bilaterian nervous systems, composing peripheral sensory structures and centralized ganglia. Interestingly, increased brain complexity appears correlated to increased glial numbers (glia compose 15% of *C. elegans* or *Drosophila* nervous systems and 50–90% of mammalian brain areas) (Freeman and Rowitch, 2013). Yet neuron and glial cell types were ill-defined by morphological criteria alone. For example, astrocytes were grouped in fibrous (stellate-shaped, with long, thin processes, predominant in white matter) and protoplasmic (with short, ramified processes, predominant in gray matter). Yet, protoplasmic astrocytes are now known to transform into fibrous astrocytes upon specific environmental or signaling cues (Sun et al., 2010). Morphological criteria alone can hamper cell classifications.

In parallel with the first morphological descriptions of neural cells, studies on nerve excitability, by Du Bois Reymond among others, pioneered early electrophysiological approaches. These provided the conceptual framework to envision circuit function as a result of electrical signals (Finkelstein, 2015). Since then, traditional electrophysiological stimulations alongside anatomical methodologies remained dominant for a half−century, extensively employed to reveal functional architecture of brain regions (Hubel and Wiesel, 1962; O’Keefe and Nadel, 1979). While focusing on electrophysiology, glial cells (from the Greek word for glue, γλ*o*íα) were described as electrically non-excitable, passive material, providing insulation and trophic support to neurons. With functions lying beyond early electrophysiological operations, glial cells were often overlooked (Varoqueaux and Fasshauer, 2017), yet they contain voltage-sensitive ion channels and neurotransmitter receptors and may exert electrical activity (Gallo and Ghiani, 2000a,b). Astrocytes and other glia interacting with axons and synapses, display a complex repertoire of Ca^2+^ signaling. The evolving field of glia neuroscience is advancing techniques for recording and studying Ca^2+^ activity, its spatiotemporal dynamics in single astrocytes and across networks (Semyanov et al., 2020; Figure 1). Today, measuring neuron and glial activities remains prominent for functional cell investigation.

### From Cell’s Molecular Content to Transcript Profiling

In the 90’s, the “decade of the brain,” electrophysiology gave ground to molecular investigations (Bargmann, 1998; Südhof and Malenka, 2008; Changeux, 2020). Hypothesis−driven experimentation steered research away from “descriptive” approaches, while the preeminent molecular biology revolution and advanced genetics in model organisms allowed for uncovering mechanisms of nervous system cell physiology and interactions (St Johnston, 2002; Rapti, 2020). Studies in invertebrates and vertebrates -spearheaded by *C. elegans*, *Drosophila*, and mice- identified conserved molecules shaping intricate cell morphologies, synaptic neurotransmission, and connectivity (Leung-Hagesteijn et al., 1992; DiAntonio et al., 1993; Nonet et al., 1993; Zallen et al., 1998). Conventionally, neurons were distinguished from glia in the basis of synaptic neurotransmission. Yet, the recently uncovered molecular signaling pathways of glia have much in common with those of neurons (Fields and Stevens-Graham, 2002; Allen and Lyons, 2018). Interestingly, work in non-Bilateria highlights that molecular components of synapses exist in animals devoid of nervous systems, such as Placozoa and Porifera, raising discussion about the exact relation between the evolutionary origins of neurons and synapses (Moroz and Kohn, 2016; Arendt, 2020). This raises the question: can synaptic molecules sufficiently define neural cell types? Challenges of early molecular classifications are more obvious in glial cells, that are transcriptionally diverse with no known universal glial markers (Zhang et al., 2014). Glial cells are recognized by immunoreactivity of the intermediate filament protein GFAP, transporters, and metabolic enzymes such as glutamine synthetase, all of them also expressed in non-neural cells (Yang and Wang, 2015).

In the last decades, the advent of transcriptomics revolutionized the molecular description of cells by high-throughput measuring of gene expression, moving away from single-gene analysis (Trapnell, 2015; Marioni and Arendt, 2017; Tasic et al., 2018). Recent transcriptomics describe the organization of cell-type landscapes in circuits of mouse, Drosophila and *C. elegans*, while whole-organism single-cell transcriptomics, first in *C. elegans* and then in the annelid *Platynereis dumerilii* and cnidarian *Nematostella vectensis*, provide pioneer insights into the molecular content of nervous system cells in Bilateria and non-Bilateria species (Cao et al., 2017; Achim et al., 2018; Loo et al., 2019; Packer et al., 2019; Taylor et al., 2021). Aside from historical classifications and alongside large-scale molecular approaches, transcript profiling was suggested as the objective approach to determining a cell’s “ground state,” the unique basis that determines the cell’s capabilities (Fishell and Heintz, 2013). Nevertheless, transcriptomics entails challenges. Neural cells are challenging to dissociate, presenting elongated processes with concomitant RNA subcellular localization, which may lead to false-negative results if disrupted during dissociation (Ho et al., 2018; Perez et al., 2021). Analyzing single-cell RNA-sequencing datasets using unsupervised clustering faces computational challenges, including difficulty to report under-represented cells (Kiselev et al., 2019). The resolution of single-cell transcriptomics distinguishes similar cell clusters, that may be states of the same “cell type.” It was suggested that no two cells are transcriptionally the same while the number of possible cell types appears proportional to the number of cells analyzed (Svensson et al., 2020). These observations emphasize the notion of cell state. Transcript variation within cell types reflects stochastic expression or responses to the environment, introducing questions of whether previously unrecognized cell states are distinct types or whether recognized types represent points in a continuum of states. Similarly to carving out research into manageable subdomains (neurodevelopment, neurophysiology), there seem to be no easy dividing lines for cell types as the organism is a continuum of spatiotemporal cell interactions.

### Following Hierarchy or Integration?

Integrating genomics with functional knowledge *in vivo* is vital for linking molecular repertoires with cell development and function. The challenge lies in defining meaningful ways to do this. Transcriptome fingerprints of cells represent genes with equal weight, but expression level is not indicative of functional impact in key cell characteristics, as discussed below. Gene-function studies distinguish cell properties that define functional identity or others that portray intrinsic variability. Some suggest that classifications should follow principal choices on “the most relevant functions” of studied cell types, but such subjective decisions may hinder discovery. Recent studies and methodologies focus on multifaceted characterization of distinct modalities of neural cell types toward integration for future multimodal classification schemes (Figure 1). The *C. elegans* neuroscience community proceeded for long in a seemingly unbiased “cataloging” of cell features (morphological, molecular, functional), which may have been a driver of continuous discovery of new cellular functions.

## Cell Classifications in the Mapped Nervous System of *Caenorhabditis elegans*

Today’s understanding of nervous systems is an amalgam of contributions of studies in invertebrate and vertebrate models. Among the most comprehensively studied nervous systems is that of *C. elegans*, the first metazoan combining organism-wide cell atlas, lineage, connectome, fully sequenced genome, whole-organism and embryo single-cell transcriptomes (Sulston and Horvitz, 1977; Sulston et al., 1983; White et al., 1986; Cao et al., 2017; Molina-García et al., 2019; Packer et al., 2019; Satterstrom et al., 2020; Taylor et al., 2021). The need for curation of a rich amount of data was met by information integration and facilitated by the limited number of *C. elegans* cells. A closer look at the multifaceted description of this system provides insights for classification schemes in more complex circuits.

**FIGURE 2:**
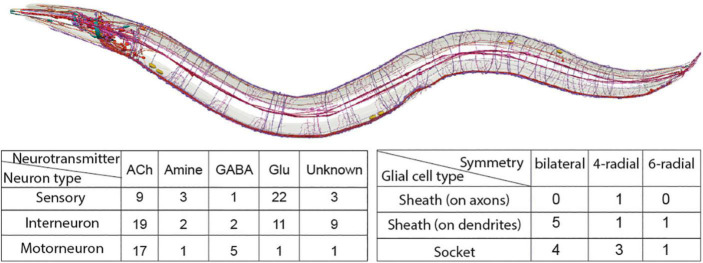
Summary of neurons (red, purple) and glial cell types (blue, cyan) in the *C. elegans* nervous system. Illustration of nervous system with neurons, glial cells and fascicles is kindly provided by Openworm.org (Sarma et al., 2018). Tables present the *C. elegans* nervous system cell types, listed based on their characterized symmetry, terminal neurotransmitter identities (for neurons), morphology (for glial cells). Sheath on axons/dendrites refers to glia with membranes that ensheath axons or dendritic tips, respectively. Details and relevant citations are provided in the text.

The *C. elegans* nervous system consists of 302 neurons and 50 ectoderm-derived glia in hermaphrodites and 387 neurons and 90 glia in males, described morphologically by pioneer studies of the first lineaging and ultrastructural analysis of an entire nervous system (Albertson and Thomson, 1976; Sulston et al., 1983; White et al., 1986; Cook et al., 2019). While its small neuron size hindered the prevalence of electrophysiology, early studies concentrated on a comprehensive mapping of neuron morphology, anatomy and connectivity at single-cell resolution and *C. elegans* neuronal cells are categorized using all these criteria combined. The *C. elegans* hermaphrodite neurons are functionally grouped into 37 sensory neurons, 44 interneurons and 23 motorneurons (Figure 2). They represent 118 neuronal classes, based on their anatomical features: 26 classes of single unilateral neurons, 70 classes of 35 bilaterally symmetrical neuron pairs, 10 classes presenting 4 radially symmetrical members, 3 classes of 6 radially-symmetrical members, 1 class with 3 head motor neuron and 8 distinct classes of nerve cord motor neurons (White et al., 1986; Hobert et al., 2016). All classes, except for the last two, include neurons of different functional modalities. Interestingly, 2 of the 70 bilaterally- symmetrical neuron pairs (AWCR/AWCL and ASER/ASEL) consist of neurons that can be further subclassified into different types due to their specific molecular diversification, as discussed below. Aside anatomy, neurotransmitter identities of all neurons are now mapped: 38 classes (78 neurons) are glutamatergic with expression of vesicular glutamate transporter EAT-4/VGLUT, 52 classes (159 neurons) are cholinergic with expression of vesicular acetylcholine transporter VAChT/UNC-17, 6 classes (26 neurons) are GABAergic expressing the biosynthetic enzyme glutamic acid decarboxylase (GAD/UNC-25), 7 classes (11 neurons) appear to be GABA-uptaking neurons expressing the vesicular GABA transporter (VGAT/UNC-47) – and 13 classes (26 neurons) are aminergic (i.e., dopaminergic, serotonergic, etc.) (Serrano-Saiz et al., 2013; Pereira et al., 2015; Gendrel et al., 2016). To date, a plethora of studies investigate these neuronal types in exquisite detail, examining mechanistically their development, specification, functions, and different states while interacting with the environment.

The *C. elegans* hermaphrodite ectoderm-derived glia, initially termed “support cells,” can be similarly classified based on anatomical features: there are 9 classes of bilaterally symmetrical pairs (ADEsh, ADEso, AMsh, AMso, OLLsh, OLLso, PDEsh, PDEso, PHsh), 5 classes of 4 radially symmetrical members (CEPsh, CEPso, OLQsh, OLQso, PHso) and 2 classes presenting 6 radially symmetrical members (ILsh, ILso) (Figure 2). Based on their anatomical relation to neurons, glia can be “sheath” glia (“sh”) or “socket” glia (so) (Ward et al., 1975; Sulston et al., 1983; White et al., 1986; Altun and Hall, 2011). “Sheath” glia present membrane processes that envelop neuronal processes, either ensheathing brain axons and synapses (CEPsh), or wrapping around dendritic endings, in sensory organs (AMsh, ILsh, OLQsh, OLLsh, PHsh). “Socket” glia in sensory organs form pores for neuronal dendritic endings to access the environment (ADEso, AMso, CEPso, ILo, OLLso, OLQso, PDEso, PHso). Several glial cells are implicated in aspects of nervous system development and function, including axon and dendrite morphogenesis, synapse positioning and neurotransmission, male-specific neurogenesis, animal longevity, locomotion, and sleep (Bacaj et al., 2008; Sammut et al., 2015; Singhvi et al., 2016; Rapti et al., 2017; Katz et al., 2018; Frakes et al., 2020). Key recognized roles of *C. elegans* glial cells are analogous to those of fly and mammalian glial cells, yet *C. elegans* glial cells remain understudied. Many of these glial cell types are not functionally characterized and how their fates are determined or compared is unknown. Whether each glial cell defines one type or multiple glia comprise the same cell type remains unknown. Notably, even for well-studied neurons, the terms “class” and “type” are used rather interchangeably, without universally sharp defining criteria. These definitions are sometimes elusive in vertebrate cell types too, and may affect cell classifications as discussed below (Tasic et al., 2018; Zhang et al., 2021).

## Cell Identity, A Multidimensional Process From Regulatory Programs to Effector Modules

Mapping the *C. elegans* nervous system anatomy and connectivity at single-cell resolution guides closely our studies of neurodevelopment and fate diversification. Regulatory programs underlying diversification suggest specific criteria for cell classification in developmental and evolutionary studies. Pioneer work in *C. elegans*, defined *terminal selectors* as master-regulator transcription factors that are continuously expressed in postmitotic cells and instruct terminal cell identity by regulating expression of cell type-specific effector genes (Hobert, 2008; Hobert and Kratsios, 2019). To date, a remarkable number of terminal selectors is identified across neuron types, highlighting a theme of combinatorial functionality (Hobert, 2016a). Strikingly, four conserved factors specify almost half of *C. elegans* neuron types while several terminal selectors are repeatedly used in distinct types (Hobert and Kratsios, 2019). For example, PROP1/UNC-42 acts as terminal selector in neurons SMD, RMD, AIB, RIV, which do not share the same neurotransmitter identity, morphology or function (SMD, RMD are motorneurons; AIB, RIV interneurons; RIV, SMD and RMD are cholinergic; AIB is glutamatergic). This is surprising at first but PROP1/UNC-42 acts with other terminal selectors in different combinations to regulate distinct fates (Berghoff et al., 2021). Remarkably, recent studies present a unique combination of homeodomain proteins expressed in each *C. elegans* neuron class, and suggest that cell type diversity can be delineated by the presence of molecular descriptors (Reilly et al., 2020). Intriguingly, terminal selectors can have different requirements across cells. Some bind DNA cooperatively, such as LHX9/TTX-3 and VSX2/CEH-10 in AIY neurons, others in an additive way like ERG/FLI1/AST-1, DLX1/CEH-43 and PBX2/CEH-20 in dopamine neurons (Altun-Gultekin et al., 2001; Wenick and Hobert, 2004; Doitsidou et al., 2013; Berghoff et al., 2021). Considering a comprehensive array of regulatory factors and their functional interactions serves better to delineate neuronal cell types than single terminal selectors alone, while experimental validation is key to define functional roles of factors in distinct cell types.

**FIGURE 3:**
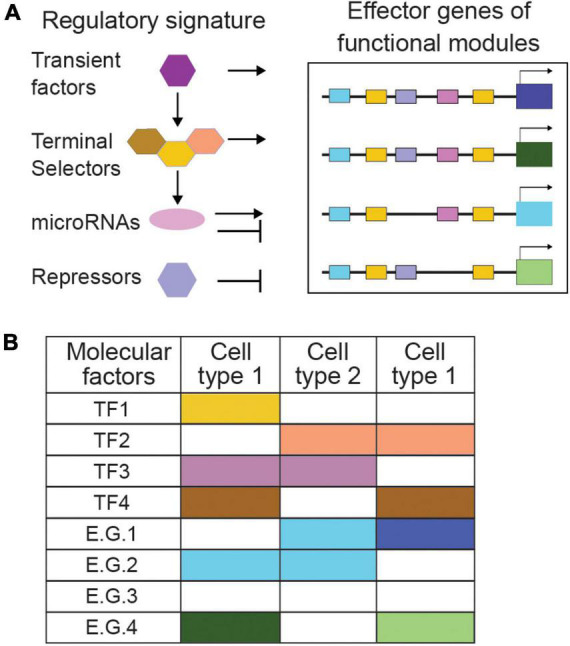
Regulatory signatures and functional modules can describe cell types by combinatorial codes. **(A)** The combination of regulatory signatures and functional modules describes cell types comprehensively. Transient factors, terminal selectors, and other activator and repressor mechanisms combined define a cell type’s ‘regulatory signature.’ **(B)** Combinatorial codes of regulatory factors direct effector genes to encode distinct functional modules in different cell types. TF, transcription factor; E.G., effector gene. Specific examples and references are provided in the text.

Alongside terminal selectors, additional mechanisms instruct neuronal and glial cell identity, including transiently expressed transcription factors (Figure 3A). Hmx/Nkx/MLS-2 regulates cell-specific expression of the terminal selector Otx/CEH-36 to control fate of AWC neurons while together with Pax6/VAB-3 it controls glial expression of the transcription factor Olig2/HLH-17 and cell development of CEPsh glia. Hmx/Nkx/MLS-2 appears only transiently expressed in embryonic AWC and CEPsh and their precursors (Yoshimura et al., 2008; Kim et al., 2010; Taylor et al., 2021). Transcriptional repressors also affect differentiation by type-specific repression of terminal selectors’ target genes (Hobert and Kratsios, 2019). Aside transcription factors, microRNAs can drive repression to define functional identity; *lsy-6* introduces asymmetry between bilateral neurons ASEL/ASER, through cell-specific repression of transcription factor NKX6.3/COG-1 while miR-791 regulates the CO2-sensing function of BAG neurons by repressing house-keeping genes (Cochella and Hobert, 2012; Drexel et al., 2016). Thus, a comprehensive repertoire of terminal selectors together with other regulatory programs compile the full array of mechanisms that control cell-specific use of genomic information, a cell type’s ‘regulatory signature’ (Arendt et al., 2016; Figure 3B).

However tempting and fruitful is to classify neural cells strictly by their regulatory signature, studying effector genes remains of paramount importance. Some transcription factors driving identity acquisition are subject to signaling by effector genes. The olfactory neurons AWCL and AWCR acquire a strikingly antisymmetric, anti-correlated fate, when correct contact of their axons triggers gap junction signaling, calcium flux, and kinase activity that feed back onto homeobox factors and microRNAs driving asymmetric gene expression and function (Hsieh et al., 2012). Thus, effector genes engage in feedback loops affecting regulatory programs. Additionally, cell-type-specific batteries of effector genes are key for identifying regulatory factors. Genetic screens for altered expression of neurotransmission effector genes uncovered the regulatory logic differentiating distinct neurotransmitter identities (Flames and Hobert, 2009; Serrano-Saiz et al., 2013; Pereira et al., 2015; Gendrel et al., 2016). Moreover, combinatorial roles of type-specific transcriptional repressors, were uncovered by following the unique expression patterns of effector genes in distinct motorneurons (Kerk et al., 2017). Delineating cell types comprehensively leans on the combination of their regulatory signature and core molecular modules of effector genes. Consequently, identifying functional genes of neural cells through *in vivo* studies remains key in nervous system investigations and classifications.

## Uncharted Terrains in Cell Types and Cell Heterogeneity

### Newly Discovered Cells Across Model Organisms

A century of cell biology and physiology would suggest that morphological and electrophysiological maps of neural cells are comprehensive in laboratory models. Yet, new cell types are still discovered in understudied and well-studied contexts. “Rosehip” GABAergic neurons, that locally control dendritic computation in pyramidal neurons, were recently discovered in layer 1 of the human neocortex (Boldog et al., 2018). In adult mouse ventricular-subventricular zones, new oligodendrocyte precursors and astrocytic cells “gorditas” were discovered upon activation of quiescent stem cells (Delgado et al., 2021). Zebrafish was thought to lack astrocytes while postembryonic radial glia were considered analogous to mammalian astrocytes in terms of gene expression and functional contribution to glutamate-dependent epileptic seizures (Lyons and Talbot, 2015; Niklaus et al., 2017). Yet, recent studies describe zebrafish cells with properties of mammalian astrocytes, such as expression of glutamate aspartate transporter, membrane tiling and association with synapses (Chen et al., 2020). Research in Drosophila discovered neurons that partition dorsal and ventral visual circuits and transient neuronal populations wrapping neuropils during development and dying before adulthood (Özel et al., 2021). Studies in *C. elegans* also present newly discovered neuron and glial cells; interneurons MCM and ciliated neurons PHD driving sexually dimorphic behavior, are generated in males by sex-shared glia AMso and PHso1 (Sammut et al., 2015; Molina-García et al., 2020). Identification of these cells was enabled by recent mapping of the nervous system anatomy and connectivity in *C. elegans* males, in contrast to the connectome of hermaphrodites already mapped for more than 3 decades (White et al., 1986; Cook et al., 2019). Besides, *C. elegans* glial cells were early mapped but only named “neuronal support cells,” yet recent in-depth functional studies uncover their glial features and analogies with vertebrate counterparts. For example, CEPsh glia are suggested to be analogous to astrocytes by molecular content and functions (Colón-Ramos et al., 2007; Yoshimura et al., 2008; Rapti et al., 2017; Katz et al., 2019). Thus, cell discovery lies in uncharted terrains of nervous systems in various, more and less complex models. As resolution in transcriptomics and functional studies increases, cell discovery continues, adding to an ongoing mapping of cell heterogeneity.

### Heterogeneity and Shared Factors Across Cell Types

**FIGURE 4:**
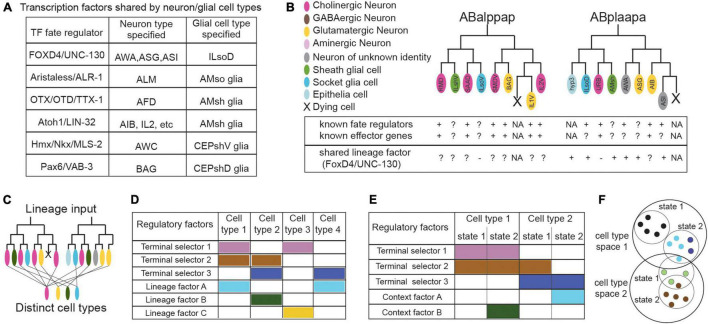
Frontiers in cell classification: regulators shared across cell types **(A)**, sparsity of known factors **(B)**, lineage convergence **(B,C)**, combinatorial profiles **(D,E)**, and the dynamics of cell states **(F)**. **(A)** Some *C. elegans* transcription factors specify both glial and neuronal cell types, while glial-specific factors are largely unknown (proteins appear as “vertebrate/*C. elegans* homolog”). **(B)** Schematics representing part of the *C. elegans* lineage and our knowledge on transcriptional regulators and effector genes of the featured cell types, that may be used as identifiers of cell identity. Known regulatory factors of identity are sometimes unknown for certain cell types, especially glial cells. The UNC-130 transcription factor regulates fate of different cell types within one sub-lineage but not similar types from convergent lineages (ILsoV, ILso). The unknowns of lineage convergence and the sparsity of known factors regulating identity can affect cell classification. Cells color-coded as indicated, gray; neurons of unknown neurotransmitter identity. Factors with functional importance; “+”, dispensable; “–”, or uncharacterized “?”, NA, non-applicable. **(C)** Schematics summarizing lineage convergence, with distinct cells arising from the same lineage and similar cell types from distant lineages. **(D)** Transcription factors have combinatorial actions for cell specification. Terminal selectors and lineage-related factors work toward diversification by lineage convergence. **(E)** Terminal selectors in conjunction with context-specific factors regulate state transitions in the same cell type. **(F)** Effector genes (encoding neurotransmitter machinery, etc.)- here in colored dots- may be shared or divergent across different states of a cell type. Single molecular identifiers may be shared between cell types, thus appearing insufficient to define them. Relevant examples and citations are provided in the text.

Neural circuit cell types were historically regarded as homogeneous cell populations, yet it becomes increasingly evident that they exhibit significant functional and molecular heterogeneity (Chaboub and Deneen, 2013; Foerster et al., 2019). A key frontline in mapping cell type heterogeneity is the biology of glial cells, their regulatory logic and divergency. Master regulators and regulatory programs of glia-specific identities often remain elusive. Early studies in Drosophila suggested that the gene *glial cells missing* (gcm) is necessary and sufficient for specification of glial cell fate (Hosoya et al., 1995; Jones et al., 1995), while later studies identified that gcm1 and gcm2 gliogenic factors also drive neurodevelopment (Chotard et al., 2005). Mammalian orthologs *Gcm1* and *Gcm2* functionally substitute for fly *gcm* but present no expression nor function in mammalian glia (Günther et al., 2000). Even in the well-studied nervous system of *C. elegans*, the regulatory logic of glial cell development is understudied, contrary to the detailed documentation of factors driving neuronal, pansensory or panneuronal identity (Swoboda et al., 2000; Stefanakis et al., 2015). Few transient transcription factors affecting glial cell identity are described. Hmx/Nkx/MLS-2 and Pax6/VAB-3 drive Olig2/HLH-17 expression in CEPsh glia, similarly to their homologs driving Olig2 expression in mouse glia, Aristaless/ALR-1 regulates the functional structure of AMso glia, FOXD4/UNC-130 instructs specification of ILsoD, Atoh1/LIN-32 instructs diversification of AMsh glia, while Prox1/PROS-1 regulates the secretome of AMsh glia and OTX/OTD/TTX-1 their stressed-induced remodeling (Tucker et al., 2005; Yoshimura et al., 2008; Procko et al., 2011; Wallace et al., 2016; Zhang et al., 2020; Mizeracka et al., 2021). Strikingly, these transcription factors regulating *C. elegans* glial fate also affect neuronal fates, alongside their glial functions (Figure 4A). MLS-2 and VAB-3 specify functional identity of AWC and BAG neurons respectively, ALR-1 ensures differentiation of touch receptor neurons, FOXD4/UNC-130 diversifies neurons AWA and ASG, TTX-1 specifies AFD neurons, while CND-1, NGN-1, and LIN-32 suppress glial fate and promote neuronal fate (Sarafi-Reinach and Sengupta, 2000; Satterlee et al., 2001; Kim et al., 2010; Topalidou et al., 2011; Brandt et al., 2019; Zhang et al., 2020). These examples of regulators shared between neurons and glial cells are often not lineage-specific (in contrast to examples of lineage convergence discussed below). In vertebrates, regulators specifying glial fates without affecting neuronal development are also sparse or lacking. Olig1 and Olig2 factors in neural progenitors drive both oligodendrocyte fate and motorneuron generation and their abolishment results in generation of interneurons and astrocytes (Anderson et al., 2002; Lu et al., 2002). Vertebrate Sox9 may promote astrogenesis by regulating the nuclear factor NFIA to maintain multipotent progenitors, while transcription factors controlling astrocyte-specific fate are unknown (Poskanzer and Molofsky, 2018). Unlike the highly methylated differentiated neurons, the mammalian glial methylome resembles the fetal methylome suggesting that glial transcriptional flexibility and heterogeneity is instructed by the environment (Poskanzer and Molofsky, 2018). Indeed, Sonic hedgehog by Purkinje neurons, drives molecular and functional diversification of Bergman glia and stellate astrocytes (Farmer et al., 2016). The quest for cell-type identifiers is ongoing even for recognised, distinct cell types.

### Sparsity of Molecular Identifiers

The sparsity of known regulators of specific fates may result from understudied functional heterogeneity (Figure 4B). Effector genes, often used as a proxy to uncover fate regulatory factors, are hardly described in glia. Besides the enzymatic apparatus driving metabolic support, glial genes driving morphogenesis and modulation of neurons are understudied, but these may have conserved roles in evolution (Heiman and Shaham, 2007). Studies dissecting *in vivo* glia development and function should provide valuable insight into their molecular repertoire of regulatory and effector genes, to guide faithful classification and cross-species study of glial cell types (Singhvi et al., 2016; Wallace et al., 2016; Rapti et al., 2017; Lee I. H. et al., 2021). Lessons from *C. elegans* studies of a modular logic for pan-neuronal fate specification suggest that pan-glial fate may also be regulated in a peace-mile manner with distinct glia types using distinct combinations of transcription factors (Stefanakis et al., 2015). Future focus on the molecular convergence and divergence of neurons and glial cells can illuminate mechanisms of neural circuit cell heterogeneity. Such challenges in uncharted terrains of cell heterogeneity across space, time, development and evolution should be taken into account when considering future schemes of investigation.

## Exploring Cell Types Throughout Nervous System Development

Current cell classifications follow the terminal neurotransmitter identity, often without involving the developmental factors that establish cell appositions underlying connectivity. This is inadvertently influenced by the prevalence of gene expression studies in postembryonic cells with defined identities. Nevertheless, should we consider regulators of development in the quest for cell type identifiers? Cell morphogenesis allows cell targeting necessary for synaptic and functional connectivity. The underlying cell-type-specific genes encoding morphogenesis factors are key features of cell physiology and undergo pressure of natural selection. Thus, morphogenesis effectors and their regulatory programs can contribute valuable information to cell type classification.

How developmental history, morphogenesis and terminal cell type function is coordinated remains elusive. During *C. elegans* brain assembly, many sister neurons present different axon paths and navigation times while some lineage-distant neurons bundle closely together (Rapti et al., 2017; Moyle et al., 2021). There are few examples of fate regulators affecting cell-type-specific morphogenesis, like the transiently expressed NKX-/MLS-2 which regulates formation of CEPsh glia and AWC neurons (Yoshimura et al., 2008; Kim et al., 2010). Recently it was demonstrated that certain connectivity features of neurons wiring together are regulated by terminal selectors (Berghoff et al., 2021). How cell-specific regulatory programs affect effector genes of morphogenesis, such as adhesion and guidance receptors, remains understudied. Mapping lineage relations, cell identities, morphogenesis and circuit functions can provide links between developmental programs and functional cell classification. This is key for a comprehensive investigation of cell types through the lens of development.

In *C. elegans*, the lineage history is invariant, was first mapped 4 decades ago (Sulston and Horvitz, 1977; Sulston et al., 1983) and can now be analyzed by automated lineage tracing (Bao et al., 2006; Boyle et al., 2006; Murray et al., 2006). Today it is extensively annotated with functional information, offering an opportunity to assess how much developmental data (lineage, state transitions, regulatory programs) are needed to meaningfully classify terminally differentiated cells and study factors of cell development in relation to cell identity. An invariant cell lineage doesn’t mean that cell fates are determined by the lineage pattern. Intriguingly, early lineaging indicated that cells with similar morphology and connectivity can be produced by distinct lineages (Sulston and Horvitz, 1977; Sulston et al., 1983). Similar lineage history appears neither necessary nor sufficient for two cells to belong to the same neuron class (Hobert, 2016b). Lineage patterns do not readily correlate with transcription factor expression, cell terminal fate, form and function. For example, the fates of six lineage-distant IL1 neurons and six lineally distant RMD neurons are specified by transcription factors SOX14/SOX-2 and PROP1/UNC-42 respectively. Multiple lineages produce highly similar neural cell types, a phenomenon termed *convergent differentiation* (Figures 4B,C). This may be explained by local inductive interactions instructing fate or shared transcription factors able to integrate distinct lineage histories (Hobert and Kratsios, 2019).

Cell type specification during development was early described in the powerful metaphor of “Waddington landscape”; cells depicted as balls traverse a hill of “epigenetic landscape” and encounter ridges or furrows that restrict their path, ultimately forcing them to stop and acquire a stable mature identity (Waddington, 1957). Conventionally, cell types were considered as monolithic points in the Waddington landscape, fixed entities with specific characteristics and features or from a systems perspective, stable fixed points in transcriptomic space. Recent transcriptomics reveals that *C. elegans* glia and neurons often become transcriptionally distinguished only in the final cell division of progenitors producing terminally differentiated cells, in contrast to non-neural tissues (muscle, dermis, intestine) which arise by lineage clades presenting within-clade transcriptomic similarity (Packer et al., 2019). Thus, neural cell types undergo a shift from lineage-correlated to fate-correlated gene expression with cells of distant lineages converging transcriptionally to adopt the same terminal fate, while diverging from their close lineage-relatives. This sudden transcriptomic shift during embryonic fate commitment of neural cells is in contrast to predictions of a smooth Waddington’s landscape. The phenomenon of convergence is not *C. elegans* specific but also prevalent elsewhere, like in mouse excitatory and inhibitory neurons (Cao et al., 2019). Overall, such dynamics of nervous system regulatory states through cell generations during development is a key challenge in developmental neuroscience.

Recent *C. elegans* studies delineating transcription factor roles in convergent differentiation in neurons or glia may provide molecular insights in other species. FOXD4/UNC-130 is expressed in and required for the diversification of different cell types (neurons AWA, ASG, ASI and glia ILsoD) arising from the same sublineage but not the diversification of similar types arising from other sub-lineages (Sarafi-Reinach and Sengupta, 2000; Mizeracka et al., 2021). On the other hand, Atoh1/LIN-32 is expressed in and required for the specification of related, left/right or radially symmetrical, neural cell types generated from distinct sublineages (Masoudi et al., 2021). The later transcription factor may control expression of terminal selectors in the specified cell types. Thus, it appears that a combination of cell type-related terminal selectors together with timely transient factors and lineage-related transcription factors underly lineage convergence and direct cell type specification (Figures 4C,D).

Combining *in vivo* studies of lineage, developmental mechanisms, molecular repertoire through transcriptomics and computational analysis will enable testable hypotheses to predict and identify links between regulatory programs of fate, morphogenesis, terminal identity, and functional connectivity. Intriguingly, cell fate specification can proceed through different pathways during natural generation of cell types or *in vitro* cell transformation induced in the laboratory (Treutlein et al., 2016). Cells derived through these different pathways are considered of the same type based on restricted molecular and morphological characteristics. Yet, how their complete repertoire of regulatory and effector genes resembles is unclear. Deciding on accepted criteria for cell-type distinctions in relation to their developmental path is important for a mechanistic understanding of cell development and function, including cell fate transformations often aiming to treat disease.

## Considering Cell States in Space and Time

Alongside cell-type heterogeneity, neural cells present spatiotemporally dynamic states, after their initial fate commitment both in complex and contained circuits. Previous studies suggest that cell states are defined by gene expression reversibly regulated by extracellular cues or transitory stimuli (Poulin et al., 2016). Well-defined criteria distinguishing cell types and states will enable to chart complex circuits lacking *in vivo* single-cell-resolution maps. Considering experimental observations in the light of current definitions can examine which sharp boundaries are delineated between cell types and states.

Several *C. elegans* neural cell types, arising from invariant cell lineages, display transcriptional changes that may underline dynamic cell states in space or time, some dependent on activity or the environment. Neural cells can undergo state changes under stress conditions. Upon starvation, the AIB interneurons change gap junction composition in response to concerted function of terminal selector PROP1/UNC-42 and the dauer-specific transcriptional regulator FoxO/DAF-16 (Bhattacharya et al., 2019). The sensory neurons IL2 remodel their dendritic architecture in response to adverse environmental conditions, also under regulation of FoxO/DAF-16 (Androwski et al., 2020). Under high temperature or starvation, the glia AMsh change morphology and undergo fusion while maintaining known fate markers. This is instructed by the GPCR/REMO-1, the transcription factor Otx1/TTX-1 and its direct target VEGFR-related tyrosine kinase FLT1/VER-1 (Procko et al., 2011; Lee I. H. et al., 2021). Then, the glia-ensheathed dendritic endings of AWC neurons also expand together with the AMsh glial membranes.

Developmental transitions also entail time-dependent cell state changes. The embryonically born DD motorneurons synaptically connect to and innervate ventral muscles, only to undergo extensive rewiring at the end of the animal’s first larval stage. In a striking example of plasticity, they eliminate early synapses and form new input and output synapses innervating dorsal muscles. Then, ventral muscles get innervated by newly born ventral VD motorneurons (White et al., 1986; Howell and Hobert, 2016; Philbrook et al., 2018). While changing circuit partners, DD motorneurons maintain their morphology and GABAergic neurotransmitter identity. This remodeling is dependent on neuronal activity, is instructed by transcription factors acting cell-autonomously and the heterochronic pathway (Hallam and Jin, 1998; Thompson-Peer et al., 2012; Miller-Fleming et al., 2016).

Cell states also occur via sex dimorphism, another context that introduces complexity in cell heterogeneity. Sexually dimorphic neurons with shared lineage and morphology present distinct gene expression, connectivity and neurotransmitter identity. During sexual maturation of males but not hermaphrodites, sex-shared interneurons AIM change neurotransmitter identity from glutamatergic to cholinergic, through a combined action of terminal selector POU4F/UNC-86 and the male-specific transcription factor LIN-29 (Pereira et al., 2015). Otherwise, the sex-shared PHB neurons undergo synaptic pruning of their juvenile synapses in interneurons AVA and AVG to maintain wiring on AVA in hermaphrodites and on AVG in males (White et al., 1986; Oren-Suissa et al., 2016; Cook et al., 2019). *C. elegans* cell state transitions may be more widespread, since gene expression is dynamic in cells across larval stages (Sun and Hobert, 2021) and appears different between embryonic and postembryonic stages of the same cells (Cao et al., 2017; Packer et al., 2019).

External signals inducing spatially or temporally distinct state transitions and their reversibility often remain elusive. Such states may have been classified as different cell types in other circuits lacking complete single-cell resolution maps. In *C. elegans* they are recognized as states of the same cell type in light of the mapped, invariant lineage and nervous system anatomy. Under the same light, bilaterally symmetric neurons AWCL/AWCR and ASEL/ASER are classified as distinct subtypes, despite sharing key regulatory factors and morphology while diverging in some effector genes and function (Hobert et al., 2016). AWCR/AWCL neurons present asymmetric expression of chemoreceptors (STR*-2* and SRSX-3 respectively) and sense different odorants (butanone and 2,3-pentanedione respectively). AWCL is considered transcriptionally the “default state,” while the alternative AWCR is generated, after induction by a transient calcium influx through voltage-gated channels and gap junctions, and downstream signaling of regulatory factors to maintain asymmetry (Alqadah et al., 2016). Similarly, bilateral neurons ASER/ASEL express distinct chemoreceptors (GCY-1,-3,-, 4-,5,-22 TRP-2 and GCY-6,-7,-14,-20, respectively) in addition to their shared receptors, and regulate different circuit outputs. Increases in NaCl concentration activate ASEL and inactivate ASER, that generate opposite intracellular Ca^2+^ transients and promote forward locomotion or reversals respectively (Suzuki et al., 2008). Each pair of AWCL/AWCR and ASEL/ASER share neurotransmitter identity and terminal selectors (OTX-1/CEH-36 or C2H2/CHE-1 respectively) but respond differently to stimuli, and this is mediated transcriptionally (Cochella and Hobert, 2012). Calcium influx acts as transient external stimuli for divergence of AWCL/AWCR, while Notch signals induce ASEL/ASER divergence (Sagasti et al., 2001; Bertrand et al., 2011; Alqadah et al., 2016). Moreover, homeotic transformations between bilateral neurons of each pair are described for both pairs (Arlotta and Hobert, 2015). Nevertheless, AWCL/AWCR and ASEL/ASER are accepted distinct types, not cell states. External cues, suggested to induce cell state changes, can often regulate divergence of cell types with distinct morphology, connectivity, regulatory and effector genes. For example, Wnt signaling through a TCF/POP-1-cascade restricts Vsx/CEH-10 expression to one of two sister cells to diversify cholinergic interneuron AIY and motorneuron SMDD (Bertrand and Hobert, 2009). Besides, fate transformations also occur elsewhere, resulting in switches between non-bilateral neuron types with distinct morphology, connectivity, neurotransmitter identity and function, like between interneurons BDU and sensory neurons ALM (Arlotta and Hobert, 2015). Consequently, cell state transitions are underlined by combined action of terminal selectors and context-specific factors and result in changing some cell-type effector genes or connectivity (Figures 4E,F). Defined stimuli or transformations alone appear insufficient to define boundaries between cell types and states; comprehensive analysis of cell properties and programs is key.

Developmental remodeling and state transitions of neural cells are observed in many circuits beyond *C. elegans*, including Drosophila photoreceptors and mammalian olfactory neurons (Sprecher and Desplan, 2008; Cheetham et al., 2016). Different neurons and glial cells in the mammalian brain exhibit graded transcriptomic differences, portraying within-cell-type heterogeneity for which neither technical nor biological noise is a likely explanation (Cembrowski et al., 2018; Tasic et al., 2018). Considering state transitions raises the question: how do regulatory mechanisms of plasticity intersect with the function of terminal selectors? The above *C. elegans* examples of context-specific cell remodeling during sexual maturation or stress, highlight an emerging theme: terminal selectors act in conjunction with condition-specific factors to induce condition-specific effector genes. Comparative single-cell transcriptomics is challenged to elucidate the extent of transient variation in a regulatory program, for example, the environmentally induced variations in cells with shared terminal selectors. Meeting this challenge is harder in complex tissues and can benefit from *in vivo* experimentation in model organisms.

## Viewing Nervous System Cell Types Through the Lens of Evolution

Incorporating evolutionary logic in the classification of neural cells is crucial in order to investigate open questions on their origins, cross-species relations and the transition from decentralized nerve nets to centralized nervous systems (Perry et al., 2017; Arendt et al., 2019; Rey et al., 2021). Differential expression of transcription factors is primarily used to build evolutionary cell-type trees. Hierarchical evolutionary classifications depict a scheme of cell diversification through genetic individuation, where a new cell type presents a new Core Regulatory Complex with at least one new transcription factor and the resulting molecular interactions (Arendt et al., 2019). Interestingly, each mature *C. elegans* neuron type expresses a unique combination of homeodomain proteins, portraying neuron type diversity, and combinatorial homeobox gene expression is also identified beyond *C. elegans* (Allen et al., 2020; Reilly et al., 2020). As discussed above, cell type specification is established by regulatory factors reused across cell types and other transient transcription factors and regulatory mechanisms. Moreover, transcript levels alone cannot always predict function of regulatory programs, as discussed below. Cell classifications serving both lenses of development and evolution would ideally incorporate known regulatory programs and functional knowledge rather than follow individual transcription factors or transcriptomic data alone. On the other hand, current neural cell classifications follow functional genes; neurons are often classified by their interneuron/sensory/motorneuron function and neurotransmitter identity (Hobert et al., 2016; Zeng and Sanes, 2017). Since natural selection acts on the fitness of animal behavior driven by effector molecules and their regulatory programs in congruence, comprehensive maps of effector genes may facilitate cell comparisons across species and mechanistic understanding of molecular diversity. Comparing entire cell transcriptomes and relative transcript enrichments is also used to delineate cell analogies across species. Relative transcript enrichment in molecular profiles of *C. elegans* glia and mouse brain cells delineates a close relationship of postembryonic CEPsh glia and mouse astrocytes (Katz et al., 2019). Such comparisons require to incorporate homologs with different number of paralogs across species, facilitated by investigating the functional importance of expressed genes.

A combined knowledge of regulatory programs and effector modules enables to trace evolution of cell types across species through the lens of both these molecular signatures combined (Arendt et al., 2016; Hobert et al., 2016; Arendt, 2020). This may allow to assess possible co-regulation of neural cell-type-specific functional modules. Evolution studies suggest that the principle neuronal characteristics, the functional molecular factors of synapses, pre-exist the origin of neurons. Modules of pre-synapse and post-synapse are separately present as modules of vesicle release, signaling and filopodia outgrowth, in non-neuronal cells of non-Bilateria organisms. Neurons may have evolved through the innovation of integration of different modules (Arendt, 2020). Whether innovation of the neuron’s origin involved a co-regulation of different neuronal modules requires further investigation. Likewise, defining the evolutionary history of glial cell types requires building a consensus for their essential functional machineries and their regulatory programs. Overall, cell type classifications incorporating definitions that enable cross-species investigations facilitate future evolutionary studies of neural cells.

## Mapping Neural Cell Types: From Single Criteria Toward a Multifaceted Classification

### Multifaceted Descriptions of Cell Types

**FIGURE 5:**
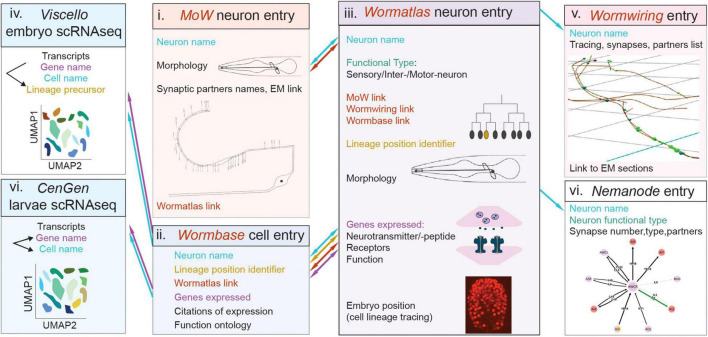
*Caenorhabditis elegans* databases allow navigation across neural cell modalities and facilitate cell classifications. Simultaneous navigation across these databases with cell entries and modalities enables certain extent of information integration, as described in the text. The schematics represents databases displaying features of cell anatomy, connectivity, molecular content. Boxes denote distinct databases. Nomenclature shared across databases, which helps integration is color-coded in blue (neuron name), orange (lineage position) or purple (gene name). Links embedded in one database, leading to another database, are color-coded in brown. Flow of navigation between databases is indicated with arrows, color-coded as described above. Morphology schematics in (i, iii) and connectivity schemes in (i, v, vii) are adapted from the named databases. Names of databases are in italics, i-vii denotes studies and databases in chronoligal sequence. Webpage links of the databases, and citations of relevant publications, are provided in the text and footnotes.

Charting the remarkable heterogeneity and cooperative roles of neural cells will pave ways toward the full picture of circuit assembly and function. Neuroscience research is moving fast toward interdisciplinary approaches to increase resolution in cell investigation. *C. elegans* is the first model with available genome sequence, lineaging, connectome and whole-organism single-cell transcriptomics. This enables nervous system mapping by molecular, anatomical and functional criteria combined, from single-cell to single-gene resolution (Figure 5). Recent breakthroughs in areas of imaging, sequencing, proteomics, and automatization enable advanced cell-type descriptions in more complex circuits as well (Figure 1). Gene profiling and electrophysiology combined, map the molecular taxonomy of mouse forebrain neurons (Sugino et al., 2006). Paired transcriptomics and proteomics investigate the molecular content of cortical neurons (Poulopoulos et al., 2019). Recent approaches allow combined electrophysiological, morphological, and transcriptomic characterization of individual neurons (Gouwens et al., 2020; Kalmbach et al., 2021; Lee B. R. et al., 2021). While early morphological classifications were considered outdated, cell morphology defines circuit function; axon appositions influence wiring and elaborate glia ramifications drive synapse ensheathment and function (Chung et al., 2015). A constantly advancing toolbox and visualization techniques highlights a come-back of morphological criteria into the picture. Light and electron microscopy reconstructions are greatly exploited for circuit mapping (Lichtman et al., 2008; Saleeba et al., 2019). Morphological reconstructions paired with high throughput electrophysiological recordings decode a wealth of morpho-electric properties (Gouwens et al., 2019). Combined expression studies and electron microscopy reconstructions in new model organisms, map tissue morphological and molecular characteristics to identify neural cell types (Vergara et al., 2021). Additionally, CRISPR/Cas9-mediated genome editing delineates neural gene function in high resolution (Nishizono et al., 2020; Fang et al., 2021). Individual researcher groups and consortia deliver large-scale profiling data and cell biology experimentalists are key to functionally dissect them. These approaches provide unparalleled resolution of a cell’s molecular content, allowing to distinguish cell clusters, hierarchical arrangement of cell populations, and transitions between states (Lähnemann et al., 2020).

### Integration of Cell Type Features

With high-throughput combinatorial approaches at hand, interpolating the anatomical, physiological, molecular and functional cell properties at single-cell-type resolution remains challenging in complex circuits. Pragmatic cell definitions following explicit, acknowledged criteria can enable investigating cell-type-specific development, function, position within taxa and ontological relations to other cells. Are single classification criteria adequate for such multifaceted investigations? Early *C. elegans* nervous system cell classifications that remain valid to date were guided by combined knowledge of the invariant cell lineage, anatomy and connectivity (Sulston and Horvitz, 1977; Sulston et al., 1983; White et al., 1986; Hobert et al., 2016). Despite its smaller cell numbers and simpler morphologies than vertebrate counterparts, *C. elegans* anatomy alone could provide sufficient resolution to distinguish most but not all recognized neural cell types. For example, neurons ASI and ASK could comprise one type based on similar axon and dendrite morphology alone but clearly constitute different types by criteria of distinct connectivity, or molecular content and functions (White et al., 1986; Taylor et al., 2021). Another primary criterion of classification is the cell’s molecular content, often represented by its transcriptome (Zeng and Sanes, 2017; Yuste et al., 2020). Yet, classifications by transcriptomics alone can present limitations. In vertebrates, cross-modal correspondence between transcriptomics and anatomy is largely strong, yet finer transcriptomic cell clusters present sometimes overlapping anatomy (Tasic et al., 2018; Yuste et al., 2020). A closer look at *C. elegans* studies suggests that transcriptomics alone cannot speak to proteins’ functional roles. LHX9/TTX-3 and LHX3/CEH-14 regulate fate of neurons AIY, ASK and neurons ALA respectively, but present different transcript levels in these neurons, sometimes not enriched (Altun-Gultekin et al., 2001; Van Buskirk and Sternberg, 2010; Serrano-Saiz et al., 2013; Taylor et al., 2021). These factors have no detected transcripts in the lineage sisters of AIY and ALA (Cengen^1^), suggesting that their relative transcript enrichment between sister cells may be more predictive of function than absolute transcript levels in a given cell. Importantly, *in vivo* expression corresponds largely well with transcriptomics. Most transcription factors specifying neuronal fates show transcripts in the neurons they specify (Hobert, 2016a; Cao et al., 2017; Packer et al., 2019; Reilly et al., 2020; Taylor et al., 2021). Yet, in few cases, factors are not clearly detected in transcriptomes of neurons that they are known to regulate. For example, LHX3/CEH-14, and Vsx2/CEH-10 specify the fate of neurons AFD and RME respectively, and are detected in these neurons by *in vivo* expression studies (Forrester et al., 1998; Serrano-Saiz et al., 2013; Kagoshima and Kohara, 2015; Gendrel et al., 2016; Taylor et al., 2021). Yet their transcripts are not clearly detected by large-scale transcriptomics probably due to incomplete profiling depth, an issue faced in single-cell-transcriptomics across organisms (Cao et al., 2017; Packer et al., 2019; Taylor et al., 2021). These comprehensive studies highlight that while “transcriptional phenotypes” show “potential” for translation, transcriptomics alone may be insufficient to predict function and expression levels adequate for protein activity vary in a given cell and process. Possible differing correlations between transcript levels and functions of cell-type molecular identifiers should be considered if classifying cell types by transcriptomics. Moreover, graded transcriptomic heterogeneity in vertebrates is widespread and functionally relevant (Cembrowski and Menon, 2018). Whether it results from within-cell-type variability or partial knowledge of cell identifiers is under investigation. *in vivo* experimentation is key to intersect trajectories of low-dimensional transcriptomic data with cell types. This does not disprove the value of anatomy and transcriptomics for classification. It highlights that finer classification is achieved when integrating them, like in *C. elegans* studies.

**FIGURE 6:**
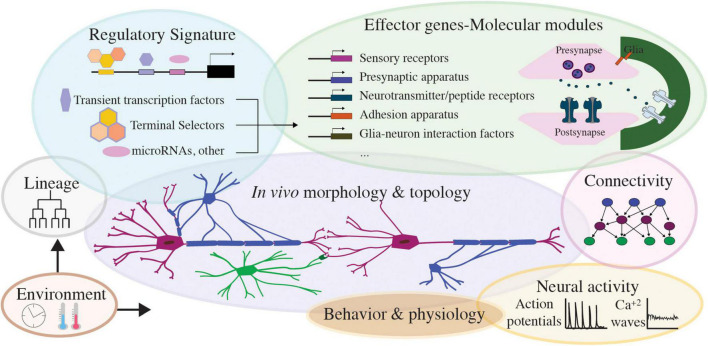
Features of nervous system cell types, toward integrated, multimodal cell classification. Schematics presenting different modalities of nervous system cell types: regulatory signature factors and core effector genes of each cell’s molecular modules, the lineage and environmental inputs influencing them, and the resulting neural cell morphology, behavior, connectivity. Activity in nervous system cells manifests as neuronal action potential or glial calcium waves and drives nervous system cell physiology and circuit function. Integrating these modalities will prove valuable toward multifaceted cell classifications across nervous systems.

Mapping uncharted circuits requires a conceptual leap linking cells’ regulatory signature and molecular make-up to functional physiology. If single criteria appear inadequate in complex circuits, hybrid approaches that consider all available information can be adapted. A useful way to classify neural circuit cells could be an integrated, multifaceted database with “cell-type spaces” presenting all features employed for classification: cell architecture, function, connectivity, lineage, regulatory and effector genes (Figures 5, 6). This inclusive cell taxonomy can depict cells as genetically encoded circuit elements, an elegant perspective to describe the brain as an organ and circuit. Early *C. elegans* nervous system classification, guided by anatomy, connectivity and mapped lineage, is in remarkable agreement with recent gene expression studies (Sulston et al., 1983; White et al., 1986; Hobert et al., 2016; Packer et al., 2019; Taylor et al., 2021). The mouse retina is another example where grouping cells by different criteria leads coherently to the same discrete neuron types (Shekhar et al., 2016). Certain features of these circuits facilitate classifications: *C. elegans* cell lineage is invariant and mapped, the retina’s laminar pattern is stereotypical and enables positional cell identification, and both have developmental patterning is seemingly ‘hard−wired’ (activity-independent). Whether such rewarding correspondence of diverse criteria will occur in other less “hard−wired” circuits with numerous cells comprising each cell type remains under investigation (Zeng and Sanes, 2017; BRAIN Initiative Cell Census Network [BICCN], 2021).

In partly mapped circuits, integrating information that feeds into morphological, molecular, wiring criteria enables harmonized cell classifications, applicable across disciplines. Like strategies in taxonomic systematics, using multiple criteria serves hierarchical classification schemes (Zeng and Sanes, 2017). Developmental and evolutionary classification emphasize regulatory programs (Arendt et al., 2019). Transcription factor combinations describing distinct cells can be identified by *in vivo* expression analysis (Reilly et al., 2020) or by computationally filtering transcriptomic cell clusters for transcription factor transcripts (Özel et al., 2021). Effector genes supporting functional modalities, like neurotransmission, also serve as primary criteria for classifications in *C. elegans*, Drosophila, mouse, and emerging-model systems (Hobert et al., 2016; Perry et al., 2017; Zeng and Sanes, 2017; Bates et al., 2019; Williams and Jékely, 2019; Özel et al., 2021). Multimodal platforms that incorporate both transcription factors and functional effectors as molecular identifiers, would enable to examine different hierarchical schemes. Understanding similarities or differences of cells depends on the information on their properties available at a given time. Each morphological, electrical, molecular or functional experimental approach detects complementary attributes. Until circuits are fully mapped, such multipurpose frameworks enable crosstalk between studies of development, function and evolution, toward a more complete image of cell types. Eventually, in well-mapped circuits, classifications by distinct criteria may greatly co-vary, like in *C. elegans* and mouse retina (Hobert et al., 2016; Zeng and Sanes, 2017).

## Feedback Loops and Multimodal Integration: Lessons From Atlases in *Caenorhabditis elegans* and Beyond

### Multifaceted Databases in *Caenorhabditis elegans*

*C. elegans* atlases provide paradigmatic platforms for cell classification. For decades, *C. elegans* cell classifications integrate morphology, connectivity and genetics in concert, and-in-hand with published and unpublished community’s knowledge. Providing ample ‘phenotypic space’ to interpolate identity and function, they afford the stereotypical map of neuron and glial cells described above. Recent transcriptomics clusters are annotated to physical cell identities by exploiting more than 868 *in vivo* expression reporters of fate and effector genes, many arising from community’s studies (Cao et al., 2017; Packer et al., 2019; Taylor et al., 2021). Matching transcriptomics to embryonic cells also utilized embryonic lineage tracing of 251 reporters (Packer et al., 2019). Transcriptomics clusters match adequately to well-studied cells, except of certain neurons (DD and VD), embryonic cells and glial cells (OLQsh, OLQso, ILsh, ILso, CEPso), understudied at single-cell resolution. Thus, community knowledge and its integration are crucial to our multimodal view of *C. elegans* neural cell types.

*C. elegans* nervous system cell classifications are organized largely in multifaceted databases, including *Wormbase^2^*, *Wormatlas^3^*, *Wormwiring^4^, Nemanode^5^*. These feature information on cell nomenclature, morphology, physiology, gene expression, wiring and display some extent of integration (Figure 5). *Wormbase* features gene entries, presenting genome location, homologies, cellular expression and function, related publications and sometimes conference proceedings. It features entries dedicated to each individual cell, recording its lineage position, reporters’ expression, citations and links to *Wormatlas*. In *Wormatlas*, webpages dedicated to each neuron present lineage identity, morphology, effector gene expression and cell function. These cell entries include links to the first connectome, the “Mind of the Worm,” (White et al., 1986; Altun and Hall, 2011) and to *Wormwiring*. *Wormwiring* presents recent matrices of process adjacencies and synaptic connections of each neural cell of both sexes (Cook et al., 2019). *Nemanode* is a recent resource of single-cell-resolution connectomes throughout *C. elegans* postembryonic development (Witvliet et al., 2021). *C. elegans* single-cell transcriptomics datasets are available in *CellAtlas^6^*, *Viscello^7^*, *Cengen^8^*. These browsers provide lists of cells presenting a specific transcript. CenGEN also provides transcript content of most nervous system cells at single-cell resolution. These platforms highlight remarkable community efforts to map nervous system cells comprehensively and dynamically. Comprehensive cell studies involve simultaneous navigation across these platforms, a feasible task owing to consistent nomenclature and limited *C. elegans* cell numbers.

These resources can be considered as graspable phases evolving toward an integrated navigable map. *Wormbase* and *Wormatlas* are scalable, continuously updated with upcoming information on cell functions and gene expression. Future curations building up on integration could enable easier navigation across different cell-specific modalities. Cell entries could integrate developmental aspects, fate regulators and embryonic physiology. They can include information on cell type/state heterogeneity, i.e., features of gene expression, process adjacencies and synaptic connectivity matrices in sex dimorphic or nutrition-deprived states (Cook et al., 2019; Witvliet et al., 2021). Including links to *Nemanode* would highlight each cell’s developmentally plastic wiring. Integrating transcriptomic profiles in *Wormatlas* or *Wormbase* entries of individual cells could enable visualizing cell-type transcripts. As glial cell studies gain considerable ground, *Wormatlas* could include entries dedicated to individual glial cells, currently missing. Embryonic cell physiology is another future frontier to tackle.

### Integration of Cell Features Beyond *Caenorhabditis elegans*

Beyond *C. elegans*, integrated platforms of multimodal “cell spaces” in different species is the current path forward to study nervous system biology in high resolution. An accumulating number of datasets, community efforts and collaborations across institutes converge toward the future picture of cell classifications. The *Fruit Fly Brain Observatory*^9^ presents fly brain neurons, their location, morphology, connectivity and biophysical properties, integrating structural and genetic data (Lazar et al., 2021). The *Allen Cell Type Database^10^* features morphological, electrophysiological features and microarray gene expression data of specific brain cells (Sunkin et al., 2013). The A*llen Mouse Brain Common Coordinate Framework i*ntegrates 3D multimodal and multiscale datasets in mouse cortical areas of 10-μm voxels (Wang et al., 2020). The *Tabula Muris^11^* compiles a compendium of transcriptomic data of mouse organs (Schaum et al., 2018). The *BRAIN Initiative Cell Census Network* (BICCN)^12^, aiming to catalog mouse, monkey and human brain cells, reported a multimodal cell census atlas of the mammalian primary motor cortex with a cross-modal analysis of transcriptomics, epigenomics, physiological and anatomical properties (BRAIN Initiative Cell Census Network [BICCN], 2021). It also provided genetic toolsets to link molecular, developmental and functional cell identities of glutamatergic projection neurons. *Hippocampome*^13^ is a comprehensive knowledge base, of 122 neuron types of the rodent hippocampus identified by literature mining based on neurotransmitter, axonal and dendritic patterns, synaptic specificity, electrophysiology, and molecular biomarkers (Sanchez-Aguilera et al., 2021). The *Human Cell Atlas*^14^ aims to map all human cells with -omics technologies (Rozenblatt-Rosen et al., 2017). Besides these multimodal sources, recent individual platforms provide data delineating mouse spatial transcriptomics of the whole brain or specific brain areas (Di Bella et al., 2021; La Manno et al., 2021). These remarkable efforts combined will enable future studies of cell type development and evolution.

### The Future of Multimodal Integration

Overall, future integrated databases can comprise cell spaces that incorporate information on lineage, physiology, morphological, electrophysiological and functional features, transcriptomics, gene-function (Figure 6). Alongside integration and mapping uncharted territories, adaptability will facilitate the dynamic improvement of these platforms. Open-access, user-accessible sources can enable personalized searches of cells based on top–down criteria, with flexibility to examine hierarchical schemes by different criteria. This would allow for testable hypothesis throughout development, across circuits, eventually across species. These platforms would involve extensive curation of information resources, and technology development for harmonizing multiple studies. They can also grow their interdisciplinarity by embracing community annotations. Early *C. elegans* transcriptomes adopted some of the first community annotation strategies, by hosting transcript cell matrices and vignettes for working with the data (Cao et al., 2017)^6^. Multimodal platforms could feature user-friendly interactive ways for experts to share feedback on the entries. They could greatly benefit from feedback-loops across disciplines, with input from experimentalists and experts in data generation and interpretation. Such feedback is critical to build up-to-date databases and refers to concepts, technologies and standardizing methods, arising from integrating multiple avenues of study. Incorporating unpublished knowledge whenever possible could accelerate the pace of scientific progress and innovation. Along the road, as the number of species with cell atlases increase, creating links between atlases of different organisms could facilitate cross-species investigations. Whether such links would follow gene homologs or specific cell modalities should be defined across communities. Integrating community atlases, -omics sources, *in vivo* experimental data and users’ feedback in a multifaceted database is the next step for comprehensive, multimodal investigations of cell types within and across species.

## Author Contributions

GR conceived the idea and composed the manuscript.

## Conflict of Interest

The author declares that the research was conducted in the absence of any commercial or financial relationships that could be construed as a potential conflict of interest.

## Publisher’s Note

All claims expressed in this article are solely those of the authors and do not necessarily represent those of their affiliated organizations, or those of the publisher, the editors and the reviewers. Any product that may be evaluated in this article, or claim that may be made by its manufacturer, is not guaranteed or endorsed by the publisher.

## Footnotes

1
https://cengen.shinyapps.io/CengenApp/

2
https://wormbase.org//#012-34-5

3
https://www.wormatlas.org/neurons/Individual%20Neurons/Neuronframeset.html

4
https://wormwiring.org/

5
https://nemanode.org/

6
https://atlas.gs.washington.edu/worm-rna/

7
https://cello.shinyapps.io/celegans/

8
https://cengen.shinyapps.io/CengenApp/

9
https://www.fruitflybrain.org/#/

10
http://celltypes.brain-map.org/

11
https://tabula-muris.ds.czbiohub.org/

12
https://biccn.org/

13
Hippocampome.org

14
https://www.humancellatlas.org/
